# Low genetic diversity, local‐scale structure, and distinct genetic integrity of Korean chum salmon (*Oncorhynchus keta*) at the species range margin suggest a priority for conservation efforts

**DOI:** 10.1111/eva.13506

**Published:** 2022-11-10

**Authors:** Ji Eun Jang, Ju Kyoung Kim, Seung‐Min Yoon, Hwang‐Goo Lee, Wan‐Ok Lee, Ji Hyoun Kang, Hyuk Je Lee

**Affiliations:** ^1^ Molecular Ecology and Evolution Laboratory, Department of Biological Science Sangji University Wonju South Korea; ^2^ Inland Aquatic Living Resources Center Korea Fisheries Resources Agency (FIRA) Yangyang South Korea; ^3^ Gyeongsangbuk‐do Freshwater Fish Research Center Uljin South Korea; ^4^ Animal Ecology Laboratory, Department of Biological Science Sangji University Wonju South Korea; ^5^ Korea Native Animal Resources Utilization Convergence Research Institute Soonchunhyang University Asan South Korea; ^6^ Korean Entomological Institute Korea University Seoul South Korea

**Keywords:** conservation, distribution limit, genetic diversity, hatchery stocking, local‐scale population structure, Pacific salmon

## Abstract

Chum salmon (*Oncorhynchus keta*) is an ecologically and economically important species widely distributed across the North Pacific Ocean. However, the population size of this fishery resource has declined globally. Identifying genetic integrity, diversity and structure, and phylogenetic relationships of wild populations of *O. keta* over an entire species' range is central for developing its effective conservation and management plans. Nevertheless, chum salmon from the Korean Peninsula, which are comprised of its southwestern range margins, have been overlooked. By using mtDNA control region and 10 microsatellite loci, we here assessed the genetic diversity and structure for 16 populations, including 10 wild and six hatchery populations, encompassing the species entire geographic range in South Korea. The analyses showed that genetic diversity is significantly higher for wild than for hatchery populations. Both marker sets revealed significant genetic differentiation between some local populations. Comparisons of six wild and their respective hatchery populations indicated that allele/haplotype frequencies considerably differ, perhaps due to a strong founder effect and/or homogenizing of hatchery populations for stocking practice. Despite its single admixed gene pool for the Korean chum salmon, some local populations housing their own unique lineages should be accorded with a high priority to safeguard their genetic integrities. The results of our comparative analyses of the Korean population with other North Pacific chum salmons (inhabiting regions of Japan, Russia, and North America) revealed a lower diversity but higher contribution to the overall species‐level genetic diversity, and also its unique genetic integrity. These findings advocate for the evolutionary significance of the Korean population for species‐level conservation.

## 1 INTRODUCTION

Salmonid fishes such as salmon, trout, chars, and graylings are widely distributed, but they are typically stenothermal cold‐water species (Reist et al., 2006; Williams et al., 2015). Salmonids are well known for their astonishing level of phenotypic variability in ecological traits, such as morphology and life history (Hendry et al., 2000; Nicieza, 1995; Taylor, 1991). Atlantic (*Salmo* spp.) and Pacific (*Onchorhynchus* spp.) salmons usually show a homing behavior, in which matured adults return to their natal rivers/streams for reproduction. The natal philopatry sometimes leads to decreasing within‐population genetic diversity, primarily due to limited gene flow occurring among geographically disconnected populations, which may ultimately accelerate the risk of local extinction (Quinn et al., 2000). Long‐distance migration behavior may also influence the extent of population differentiation and structuring of salmonid species, sometimes causing genetic isolation among local populations (Dingle, 1991; Wade & McCauley, 1988). Several previous studies on salmonid species have documented that anadromous behavior has significant effects on within‐population genetic diversity, the level of gene flow, and also effective population sizes (*N*_e_) of wild populations (Adkison, 1995; Consuegra et al., 2005; Neville et al., 2006). In some cases, genetic isolation or genetic homogeneity among wild and hatchery populations might result from unintentional anthropogenic impacts for fishery resource management (Östergren et al., 2021; Ozerov et al., 2016; Saint‐Pé et al., 2018). Climate change can also negatively affect salmonid genetic variability followed by a collapse of wild populations (Kovach et al., 2012; Valiente et al., 2010).

In fisheries, salmons have been particularly preferred for stocking and have been exploited worldwide for sustainable commercial and recreational (cultural) resources. Goals of captive‐releasing programs include enhancing depressed wild populations in numbers, establishing new populations in nonindigenous habitats if necessary, and supplementing the number of individuals for the avoidance of local extinction (Laikre et al., 2010). The use of hatchery‐reared fish, on the other hand, focused on the stock enhancement to replenish the abundance of fishery resource and restore depleted spawning biomass (Bell et al., 2008). Stocking as an effective tool for fishery management, however, has been controversial (Araki & Schmid, 2010). Potential genetic problems have been highlighted by some salmonid studies that found adverse fitness effects of introgression of maladapted genes of hatchery populations. The negative influences involve with the replacement of indigenous gene pool on adaptability of wild populations, as adaptation potential differs between hatchery (or stocked) and wild population environments because of differing selection pressures (Araki & Schmid, 2010; Harrison et al., 2018; Utter, 1998).

**FIGURE 1:**
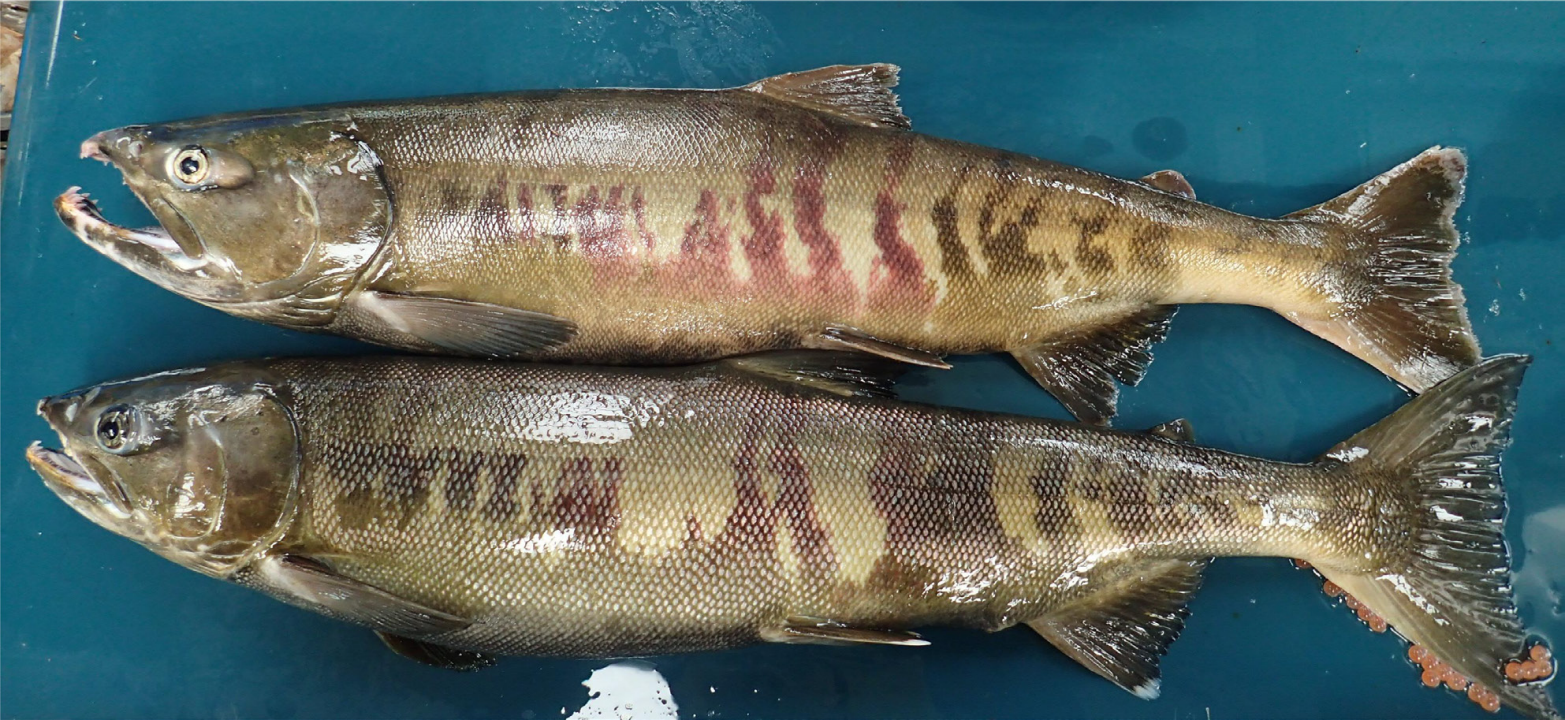
Photo of adult male (above) and female (below) chum salmon individuals caught in the Yangyang Namdae Stream (YNw) from South Korea.

**FIGURE 2:**
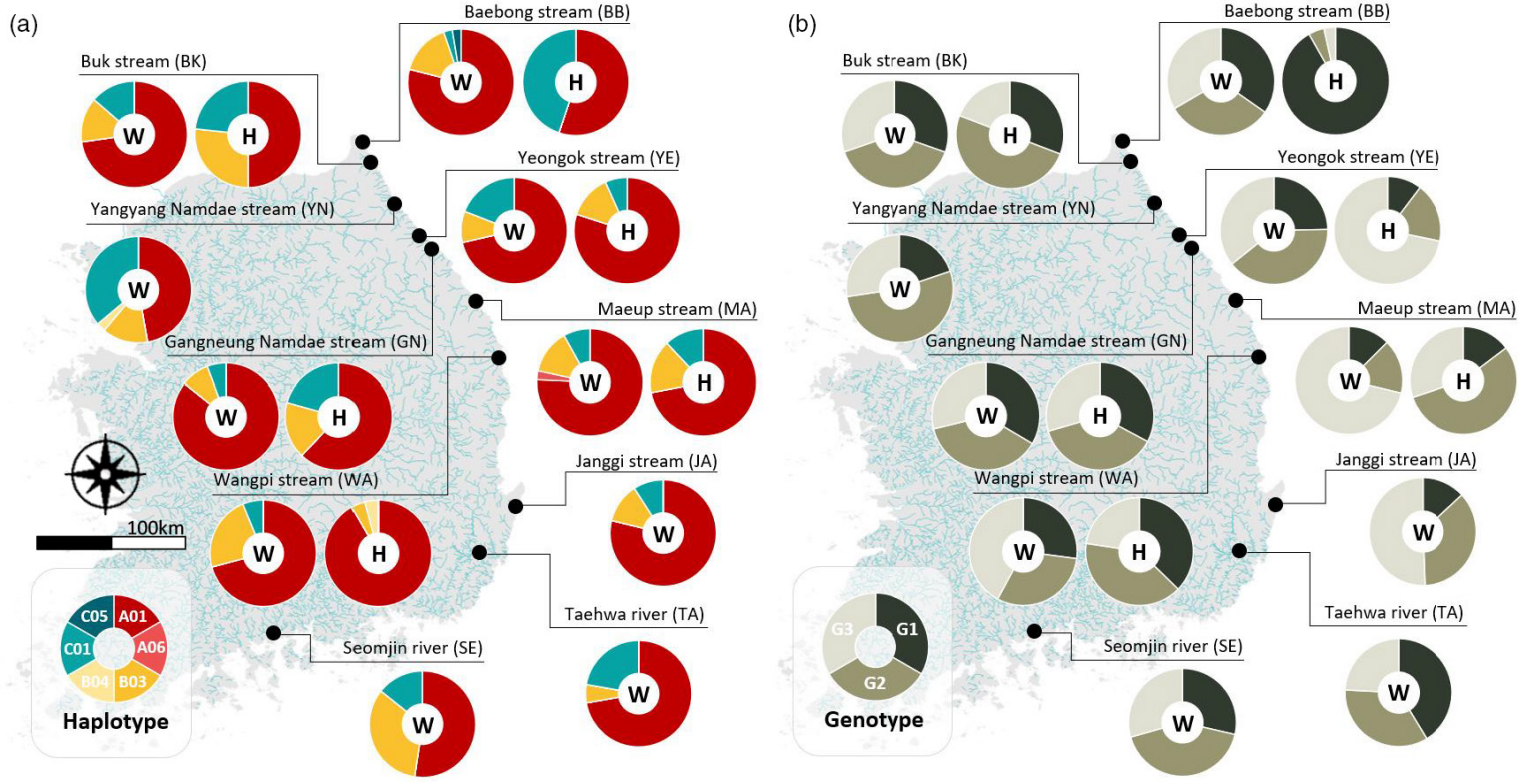
Geographic distribution of mtDNA control region (CR) haplotypes (a) and microsatellite genotypes (b) from 16 sampling populations (including 10 wild and six hatchery samples) in South Korea. The alphabets W and H in white circles denote wild and hatchery populations, respectively.

Pacific salmon is an ecologically and economically important genus for several regions particularly surrounding the North Pacific Ocean including from Northeast Asia to North America. The North Pacific Anadromous Fish Commission (NPAFC) has regularly monitored and managed the annual commercial catches and hatchery releases during the last few decades. Only less than 1% of the annual catch of salmon was recorded from Korea (NPAFC, 2018, 2019). Chum salmon (*Onchorhynchus keta*) in the Korean Peninsula, comprising the southernmost limit of its geographical distribution, migrate over long distances through Russia and Alaska to the Oregon of North America (Salo, 1991). The Korean chum salmon populations have undergone a relatively short history of hatchery‐rearing and wild‐releasing practice compared to other geographic regions. Since the Inland Aquatic Living Resources Center was established from the beginning of the 1980s in Korea, considerable efforts have been made every year, such as collecting mature eggs and artificial insemination from returning adult salmons and releasing juvenile individuals into approximately 20 streams in wild along the East Coast of Korea (Kim, Lee, & Kang, 2007). For the artificial fertilization in hatcheries for the restoration program, matured females and males returning to the Yangyang Namdae Stream (YNw) (Figure 1), which is comprised of the largest population in Korea, were mainly used for establishing the seeds for stocking. For developing effective fishery managements of chum salmon, a number of population genetic studies using molecular markers have been conducted on natural populations in different geographic regions (Japan: Beacham, Sato, et al., 2008; Sato et al., 2001; Tsukagoshi et al., 2016, Russia: Afanas'ev et al., 2011; Efremov, 2001, North America: Olsen et al., 2008; Petrou et al., 2014; Small et al., 2015). Only a handful of studies have genetically evaluated the entire population of *O. keta* across the North Pacific Ocean (mitochondrial DNA [mtDNA]: Kim, Lee, Kang, Kim, et al., 2007; Sato et al., 2004; Yoon et al., 2008, microsatellites: Beacham, Sato, et al., 2008; Beacham, Varnavskaya, et al., 2008; Beacham et al., 2009; single nucleotide polymorphisms [SNPs]: Seeb et al., 2011). Unfortunately, however, only one to at most three Korean populations were included in those studies. This would be an oversight particularly given the Korean population is at the equatorial margin of the geographical range of the chum salmon. Recently, the results of microsatellite analysis on seven populations (Myeongpa, Buk, Namdae, Yeongok, Maeup/Osip, and Wangpi Streams and Taehwa River; Figure 2) occurring on east flowing rivers in Korea have been reported in NPAFC (Kim & Kim, 2019), but more an in‐depth evaluation with adding more populations, larger sample sizes, and more markers is needed. Genetic structure of hatchery populations in Korea also remains unexplored.

In this study, we investigated the local‐scale genetic diversity and population structure of chum salmon, *O. keta* spanning nearly an entire geographical range from South Korea along the East Sea coastlines, including 10 wild and six hatchery‐reared populations. The goals of this study are (1) to analyze and compare the level of genetic diversity between wild and hatchery populations; (2) to estimate the level of genetic differentiation and structure among/within Korean wild and hatchery populations; (3) to identify the level of genetic admixture in wild populations introduced from hatchery stocks; (4) to infer the phylogenetic relationships among the entire North Pacific populations of *O. keta*, with a focus on the Korean clades. By analyzing and contrasting the levels of genetic diversity and divergence of the Korean population to those of other regional populations, we further discuss on the importance of Korean chum salmon population for an entire species‐level conservation. Our findings inform on conservation implications for salmon fishery resource in order to establish future effective management plans, for example, not only designing appropriate captive‐release programs in Korea, but also a whole species‐level global plan for its conservation.

## 2 MATERIALS AND METHODS

### 2.1 The study populations and sampling localities

A total of 537 samples of *O. keta* (for mtDNA: *N* = 516; microsatellites: *N* = 454) were collected from 10 wild populations and six hatcheries from South Korea between February 2017 and March 2019 (Tables 1 and 2; Figure 2). Wild‐caught salmon specimens (*N* = 364) were obtained from returning adults during a spawning period of October to November in 2017–2018 years, and also collected in the following winter or early spring (February to March) as fry and/or juveniles (standard length: 2.1~5.8 cm, weight: 0.1~1.8 g) using skimming nets (Table 1). The wild‐caught samples were comprised of individuals from: the Baebong Stream (BBw; *N* = 38), Buk Stream (BKw; *N* = 44), Yangyang Namdae Stream (YNw; *N* = 36), Yeongok Stream (YEw; *N* = 21), Gangneung Namdae Stream (GNw; *N* = 35), Maeup Stream (MAw; *N* = 37), Wangpi Stream (WAw; *N* = 52), Janggi Stream (JAw; *N* = 33), Taehwa River (TAw; *N* = 37), and Seomjin River (SEw; *N* = 31) (Table 1; Figure 2). For wild populations, a small piece (<3 mm) of fin tissues was collected from adipose and caudal fins for adults and juveniles, respectively, and then released immediately back to the original sites. Hatchery individuals (*N* = 173) were obtained from three hatchery facilities: Korea Fisheries Resources Agency (FIRA), Gyeongsangbuk‐do Freshwater Fish Research Center (GFFRC), and Samcheok Aquatic Fisheries Resource Center (SAFRC). The FIRA, which is a major hatchery center located at the Yangyang Namdae Stream (YN), runs hatchery‐rearing and wild‐releasing practice for salmons that return to several natal streams, including the Baebong Stream (BBh; *N* = 29), Buk Stream (BKh; *N* = 18), Yeongok Stream (YEh; *N* = 30), and Gangneung Namdae Stream (GNh; *N* = 30), SAFRC the Maeup Stream (MAh; *N* = 34), and GFFRC the Wangpi Stream (WAh; *N* = 32; Table 1; Figure 2). Hatchery individuals for each stream were reared from artificially incubated fertilized eggs bred by artificial insemination with sperm and eggs of wild male and female individuals sourced from the same stream at a ratio of 5:5. The hatchery‐reared juvenile fish from four natal streams (BBh, BKh, YEh, GNh) ran by the FIRA were customarily admixed and released back into each natal stream in the upcoming winter or early spring, while those hatchery‐reared fish descending from MAh and WAh were only released into each of their natal rivers, MAw and WAw, respectively. Alternatively, five streams (BBw, BKw, YNw, YEw, and GNw) were primarily stocked (approximately 70% of pooled hatchery juveniles) with hatchery‐reared juveniles descending from the Yangyang Namdae Stream (YNw), while the remaining 30% were hatchery juveniles sourced from the other four streams (BBw, BKw, YEw, and GNw) at about an equal ratio of 1:1:1:1. All hatchery samples used in this study were obtained before being admixed, which indicates that in the case of hatchery samples for BBh, BKh, YEh, and GNh, they were taken prior to admixing. Moreover, wild fry and/or juvenile samples were collected prior to the releasing (stocking) practices to avoid the presence of hatchery fish in the wild‐caught samples (Table 1). All tissue samples were individually preserved in 99% ethanol and stored at 4°C until genomic DNA extraction was undertaken with DNeasy Blood & Tissue Kit (Qiagen).

**TABLE 1:** Information of samples of *Oncorhynchus keta* from 16 populations (including 10 wild and six hatchery populations) from Korea

Population	Coordinates	Classification	Abbreviation	*N*_T_	Year of collection				
					2017		2018		2019
					*N*_FJ_	*N*_ADT_	*N*_FJ_	*N*_ADT_	*N*_FJ_
Baebong stream	38°32′14.84″N 128°24′11.09″ E	Wild	BBw	38	‐	‐	‐	26	12
		Hatchery	BBh	29	‐	‐	‐	‐	29
Buk stream	38°23′13.67″N 128°27′40.95″ E	Wild	BKw	44	25	‐	‐	12	7
		Hatchery	BKh	18	‐	‐	‐	‐	18
Yangyang Namdae stream	38°04′56.06″N 128°38′17.14″ E	Wild	YNw	36	26	‐	‐	10	‐
Yeongok stream	37°50′52.50″N 128°48′52.67″ E	Wild	YEw	21	21	‐	‐	‐	‐
		Hatchery	YEh	30	‐	‐	‐	‐	30
Gangneung Namdae stream	37°45′24.65″N 128°54′24.53″ E	Wild	GNw	35	‐	‐	‐	27	8
		Hatchery	GNh	30	‐	‐	‐	‐	30
Maeup stream	37°22′44.66″N 129°13′43.90″ E	Wild	MAw	37	‐	‐	‐	29	8
		Hatchery	MAh	34	‐	‐	34	‐	‐
Wangpi stream	36°57′55.30″N 129°23′33.40″ E	Wild	WAw	52	‐	29	‐	15	8
		Hatchery	WAh	32	‐	‐	32	‐	‐
Janggi stream	35°53′52.58″N 129°30′52.99″ E	Wild	JAw	33	‐	‐	‐	25	8
Taehwa river	35°33′39.75″N 129°14′59.29″ E	Wild	TAw	37	‐	‐	‐	30	7
Seomjin river	35°11′24.86″N 127°32′46.90″ E	Wild	SEw	31	‐	‐	‐	31	‐
Total				537	72	29	66	205	165

Abbreviations: *N*_ADT_, number of samples analyzed for returning adults caught between October and November; *N*_FJ_, number of samples analyzed for fry and/or juvenile salmons caught between February and March; *N*_T_, total number of samples analyzed.

**TABLE 2:** Diversity indices of *Oncorhynchus keta* at mtDNA control region (CR) and 10 microsatellite loci

Abbreviation	MtDNA control region					Microsatellites								
	*N*_MT_	*N*_H_	*H*	π	HR	*N*_MS_	*N*_A_	AR	PA	*H*_E_	*H*_O_	*F*_IS_	HWE	Median of *N*_e_
BBw	38	4	0.360	0.002	2.101	26	13.7	10.7	2	0.884	0.759	0.144	<0.001	1233.2 (101~∞)
BKw	44	3	0.444	0.002	1.971	32	16.2	11.4	4	0.890	0.852	0.044	<0.001	35.2 (25.8~51.2)
YNw	36	4	0.644	0.003	2.575	36	17.4	11.7	5	0.895	0.852	0.048	<0.001	245.7 (95.5~∞)
YEw	21	3	0.467	0.002	2.000	21	14.2	11.6	2	0.882	0.852	0.034	<0.001	79.8 (37.3 ~ 6222.7)
GNw	35	3	0.262	0.001	1.791	25	14.8	11.2	3	0.876	0.809	0.079	<0.001	45.4 (26.6~112.1)
MAw	37	4	0.413	0.002	2.485	20	12.6	10.0	5	0.854	0.732	0.146	<0.001	infinity (81.7~∞)
WAw	48	3	0.451	0.003	1.830	34	16.0	11.0	4	0.869	0.744	0.145	<0.001	599.1 (144.6~∞)
JAw	33	3	0.367	0.002	1.948	20	11.7	9.9	3	0.870	0.758	0.131	<0.001	437.4 (55.9~∞)
TAw	36	3	0.438	0.001	1.833	30	15.9	11.5	3	0.885	0.786	0.114	<0.001	infinity (442.8~∞)
SEw	21	3	0.624	0.003	2.000	28	16.2	11.8	1	0.890	0.810	0.091	0.0025	304.9 (78.1~∞)
All wild	349	6	0.454	0.002	2.053	272	26.3	11.1	60	0.890	0.884	0.097	<0.001	‐
BBh	29	2	0.512	0.001	1.000	29	9.0	7.8	2	0.825	0.884	−0.073	0.0048	16.8 (11.5~25.9)
BKh	30	3	0.646	0.003	2.000	29	15.6	11.3	10	0.895	0.854	0.046	<0.001	168.2 (71.2~∞)
YEh	30	3	0.349	0.002	1.913	29	12.5	9.5	3	0.844	0.809	0.043	<0.001	18.1 (13.1~26.1)
GNh	29	3	0.562	0.003	1.999	30	11.7	9.2	3	0.825	0.744	0.099	<0.001	26.9 (18.4~43.8)
MAh	25	3	0.460	0.002	1.998	33	14.0	10.3	2	0.871	0.816	0.065	<0.001	30.8 (21.6~47.6)
WAh	24	3	0.163	0.001	1.750	32	14.7	10.6	4	0.869	0.842	0.032	0.0004	59.9 (36.4~133.1)
All hatchery	167	4	0.493	0.002	1.777	182	23.0	9.8	27	0.804	0.826	0.066	<0.001	‐
Total	516	6	0.466	0.002	‐	454	29.2	‐	‐	0.889	0.809	0.090	<0.001	‐

*Note*: A critical threshold value for the lowest allele frequency (P_crit_) of 0.05 was used for the LD‐based *N*_e_ estimations, and point estimates (median of *N*_e_) and associated 95% jackknife confidence intervals (95% CI) are given.

Abbreviations: AR, allelic richness; *F*_IS_, inbreeding coefficient; *H*, haplotype diversity; *H*_E_, expected heterozygosity; *H*_O_, observed heterozygosity; HR, haplotype richness; HWE, *p* values for multilocus tests for Hardy‐Weinberg Equilibrium; Median of *N*_e_, contemporary effective population sizes estimated based on linkage disequilibrium (LD) method using NeESTIMATOR v2.01 (Do et al., 2014); *N*_A_, observed mean number of alleles across 10 loci; *N*_H_, number of haplotypes; *N*_MS_, number of samples analyzed for microsatellites; *N*_MT_, number of samples analyzed for mtDNA CR; PA, number of private alleles; *π*, nucleotide diversity.

### 2.2 Mitochondrial DNA sequencing

MtDNA control region (CR; 599 bp) was amplified by polymerase chain reaction (PCR) using tRNAthr‐2 and tRNAphe‐2 primers (Sato et al., 2001) with following reactions, comprised of 25 μM of each dNTP (Bio Basic), 0.6 μM each of the forward and reverse primers, 0.2 U *Taq* DNA polymerase (Thermo Fisher Scientific), 1× PCR buffer, and approximately 10–40 ng of template DNA for a final volume of 15 μl. The amplified PCR products were purified enzymatically with Exonuclease I and Shrimp Alkaline Phosphatase (New England BioLabs). The purified mtDNA CR fragments were sequenced in a single forward direction using an ABI 3730xl Genetic Analyzer (Applied Biosystems) at the Humanizing Genomics Macrogen in Korea. The obtained DNA sequences were edited using GENEIOUS v11.1.5 (Kearse et al., 2012), and then manually verified.

### 2.3 Microsatellite genotyping

Ten microsatellite loci were amplified for every individual with published primers for family Salmonidae [*Oncorhynchus keta* (Oke3: Buchholz et al., 2001; chums117, chums134: Tsukagoshi et al., 2015), *Oncorhynchus mykiss* (Omy1011: Spies et al., 2005), *Oncorhynchus nerka* (One101, One 102, One 104, One111, One 114: Olsen et al., 2000), *Salmo salar* (Ssa419UOS: Cairney et al., 2000)]. The eight microsatellite markers (except chums117 and chums134) were chosen based on previous studies of *O. keta* (Beacham et al., 2009; Beacham, Sato, et al., 2008). A M13 universal primer labeled with fluorescent dyes, such as 6‐FAM, HEX, TET, or ATTO565 was used. PCR reactions were performed using the same method as mtDNA (see above). A 1.5 mM of MgSO_4_ (Bio Basic) were added to PCR reactions for the markers Omy1011, One102, and One114. The thermocycler conditions were consisted of 94°C for 10 min, 35 cycles at 94°C for 30–40 s, 50–65°C for 30–40 s, and 72°C for 30–40 s and a final extension step at 72°C for 5–20 min. Following PCR reactions, samples were electrophoresed on an ABI 3730xl automated DNA sequencer (Applied Biosystems). Fragment sizes were determined with the ROX 500 bp size standard (ABI) using GENEMAPPER software v5.0 (Applied Biosystems) at the Humanizing Genomics Macrogen. A single person (JEJ) carried out allele scoring for 10 loci and checked for an error with Microsatellite Toolkit in MS Excel (Park, 2008).

### 2.4 Data analyses

#### 2.4.1 Genetic diversity within Korean populations

For mtDNA CR, the number of haplotypes (*N*_H_), haplotype diversity (*H*), and nucleotide diversity (*π*) were estimated for each population and the entire pooled Korean *O. keta* population using ARLEQUIN v3.5 (Excoffier & Lischer, 2010). The rarefaction method was applied using CONTRIB v1.02 (Petit et al., 1998) to calculate haplotype richness (HR), corrected for unequal sample sizes across the 16 populations of *O. keta*.

For 10 microsatellite loci, mean number of alleles per locus (*N*_A_), allelic richness (AR) corrected for unequal sample sizes, number of private alleles (PA), observed (*H*_O_) and expected (*H*_E_) heterozygosity, inbreeding coefficient (*F*_IS_), and Hardy–Weinberg equilibrium (HWE) statistics were calculated using GENEPOP v4.0 (Rousset, 2008) and FSTAT v2.9.3.2 (Goudet, 2001). The frequencies of null alleles were estimated across the 10 loci to test for the reliability of microsatellite genotyping using GENEPOP. We found that the null allele frequencies ranged from 0.012 (chums117) to 0.107 (One111) (mean = 0.041), indicating a very low probability of null alleles present in our dataset.

To examine whether there were significant differences in the level of genetic diversity between wild (*N* = 10) and hatchery (*N* = 6) populations, HR, AR, *H*_E,_ and *H*_O_ values were statistically analyzed by each separate independent *t*‐test. Contemporary effective population sizes (*N*_e_) were also calculated for each of the samples based on linkage disequilibrium (LD) method implemented in NeESTIMATOR v2.01 (Do et al., 2014). A critical threshold value for the lowest allele frequency (P_crit_) of 0.05 was used for the *N*_e_ estimations, and 95% confidence intervals (95% CI) were derived from jackknifing across all loci. Difference in *N*_e_ estimates between wild (*N* = 8) and hatchery (*N* = 6) populations excluding the MAw and TAw populations with infinite of *N*_e_ estimates (Table 2) was analyzed by a nonparametric Mann–Whitney *U* test.

#### 2.4.2 Genetic differentiation and structure among Korean populations

Level of genetic differentiation was evaluated within and between wild and hatchery samples using pairwise estimates of *F*_ST_ with haplotype (mtDNA) and allele (microsatellites) frequencies using ARLEQUIN and GENEPOP. For the three wild populations (BKw, YNw, and WAw), we performed temporal analyses of *F*_ST_ statistics over 3 years by pooling returning adults in the fall (October to November) and fry and/or juveniles in the following winter or spring (February to March; 2017FJ vs. 2017ADT & 2018FJ vs. 2018ADT & 2019FJ; Table 1) before pooling the samples. The significance of multiple comparisons was evaluated after the Bonferroni correction at a global significance level of α = 0.05.

Population structure and genetic admixture were inferred using STRUCTURE v2.3.1 (Pritchard et al., 2000), which uses Bayesian clustering of multilocus genotypes to assign individuals to particular *O. keta* populations. To obtain a representative value of genetic clusters (*K*) in the data set, we ran 10 independent runs at each *K* = 1–10 (considering only 10 wild populations) with a burn‐in length of 10^5^ followed by 10^6^ replicates of Markov Chain Monte Carlo (MCMC). The distribution of the delta *K* based on the rate of change in the log probability of data Ln *K* between successive *K*‐values (the best *K* shows the highest delta *K*‐value; Evanno et al., 2005) was estimated using STRUCTURE HARVESTER website (Earl, 2012).

To test if the genetic structure exists between wild and hatchery samples, we analyzed the whole data set by including wild and hatchery populations together using the same STRUCTURE settings as above by assuming *K* = 1–16. Moreover, a principal component analysis (PCA) was performed to assess genetic relationships among populations with multilocus microsatellite genotypes using GenAlEx v6.502 (Peakall & Smouse, 2006). A hierarchical analysis of molecular variance (AMOVA) was further performed using ARLEQUIN to test for the population structure between wild and hatchery populations. Two different AMOVAs were run to estimate the source of variation found between wild and hatchery groups (*F*_CT_), among populations within groups (*F*_SC_), and within populations (*F*_ST_) for the mtDNA and microsatellites separately with 10,000 permutations.

In addition, to test if temporal variation exists in the genetic structure among the sampling years, temporal AMOVAs were analyzed at both mtDNA and microsatellites by grouping all wild populations according to the sampling years (e.g., 2017FJ vs. 2017ADT & 2018FJ vs. 2018ADT & 2019FJ; Table 1).

Spatial patterns of genetic discontinuity were visualized using BARRIER v2.2 (Manni et al., 2004) using coordinates and pairwise *F*_ST_ values, based on the Monmonier's maximum difference algorithm, in order to determine whether genetic discontinuity exists across wild populations. Additionally, we tested for isolation by distance (IBD) among wild populations based on *F*_ST_ values using the Mantel test in GENALEX v6.502. The geographic surface distance in kilometers between two sampling sites was acquired from the website (http://www.movable‐type.co.uk/scripts/latlong.html).

#### 2.4.3 Large‐scale phylogenetic/genetic comparisons among North Pacific populations

To infer large‐scale phylogenetic relationships among CR haplotypes of *O. keta* distributed in the North Pacific Rim, we used the previously registered 30 sequences [GenBank Accession Nos.: AB039890–AB039901 (Sato et al., 2001), AB091514–AB091531 (Sato et al., 2004), AB524890 (unpublished; Table S1)], which were found to be grouped into three major clades A, B, and C, based on 20 SNPs in the CR partial segments (481 bp) in 2162 individuals from 48 populations in the Pacific Rim [Korea (number of populations: *N* = 1), Japan (*N* = 16), Russia (*N* = 10), North America (*N* = 21)] (Sato et al., 2004). The six haplotypes determined in this study (GenBank Accession Nos. ON989345–ON989350) were adjusted to a shorter length of 481 bp. All the 30 sequences including our determined six haplotypes were aligned, edited in GENEIOUS and haplotype network was then reconstructed using HAPSTAR v0.7 (Teacher & Griffiths, 2011) to determine the global‐scale phylogenetic relationships among the mtDNA haplotypes, with a focus on relative proportions of the three haplotype lineages (A, B, and C) that each population represented.

To determine the level of genetic diversity and genetic integrity of Korean populations, we analyzed and compared the genetic diversity and differentiation among the entire North Pacific Ocean populations. These analyses excluded hatchery samples and included only wild populations returning to their natal rivers. A total of 2511 fishes were analyzed for the mtDNA CR, including Korean populations (*N* = 349) from this study, Korean (YNw; *N* = 46), Japanese (*N* = 731), Russian (*N* = 406), and North American populations (*N* = 979) analyzed in Sato et al. (2004). As sample sizes varied among the four regional populations, CONTRIB was used to standardize unbalanced sample sizes, and haplotype frequencies were then calculated to estimate *H*, HR, divergence of regional populations (*H*_S_, *H*_T_, *G*_ST_) and contribution to total diversity (*C*_T_, *C*_S_, *C*_D_) and total haplotype richness ($C_{T}^{r}$, $C_{S}^{r}$, $C_{D}^{r}$). According to Petit et al. (1998) and Quan et al. (2021), this method assesses the total genetic diversity a population contributes when added to all other populations (*C*_T_) which is broken down into the genetic variation contribution (*C*_S_) and the genetic distinctiveness contribution (*C*_D_) of the population. Similarly, the total haplotype richness contributed by each population to the while ($C_{T}^{r}$) is broken down into the haplotype variation contribution ($C_{S}^{r}$) and the haplotype distinctiveness contribution ($C_{D}^{r}$). Pairwise population differentiation among the four North Pacific populations was estimated using *F*_ST_ statistics computed from ARLEQUIN. We also compared the microsatellite diversity across the same marker‐set of eight out of 10 microsatellites (Oke3, Omy1011, One101, One102, One104, One111, One114, Ssa419UOS) analyzed in common between previous (Beacham et al., 2009) and current studies. Unfortunately, however, only *N*_A_ across the eight loci could be calculated for the four regional populations (number of population; Korea: *N* = 10; Japan: *N* = 7; Russia; *N* = 8; North America: *N* = 13).

## 3 RESULTS

### 3.1 Genetic diversity within Korean populations

Only six mtDNA CR haplotypes (599 bp) were found in 516 chum salmon individuals (A01, A06, B03, B04, C01, C05; Figure 2a). Each population had two to four haplotypes belonging to three North Pacific lineages (A, B, and C), although BBh had only two haplotypes (A01, C01) and WAh had no haplotypes belonging to lineage C. While wild populations had all the six haplotypes, hatchery populations had only four haplotypes except for haplotypes A06 and C05, which were observed only in MAw and BBw, respectively (Figure 2a). The overall *H* was 0.466 ± 0.023, and *π* was 0.002 ± 0.002 (Table 2). The highest and second highest levels of *H* values were identified in BKh (0.646) and in YNw (0.644), and the lowest in WAh (0.163). HR values ranged from 1.791 (GNw) to 2.575 (YNw) for wild populations and from 1.000 (BBh) to 2.000 (BKh) for hatchery samples.

For microsatellites, 454 chum salmons from the entire population had a *N*_A_ of 29.2 across the 10 loci, which was shown to be divided into three genetic clusters (Figure 2b). *N*_A_ ranged from 9.0 (BBh) to 17.4 (YNw), AR from 7.8 (BBh) to 11.8 (SEw), and PA from 1.0 (SEw) to 10.0 (BKh) (Table 2). Entire pooled wild population showed that *H*_E_ and *H*_O_ values were 0.890 and 0.884, respectively with *F*_IS_ = 0.097, indicating significant deviation from HWE. Hatchery populations also revealed that *H*_E_ and *H*_O_ values were 0.884 and 0.826, respectively, with *F*_IS_ = 0.066. These findings suggest that nonrandom mating, most likely inbreeding, is occurring within both of the wild and hatchery populations.

**FIGURE 3:**
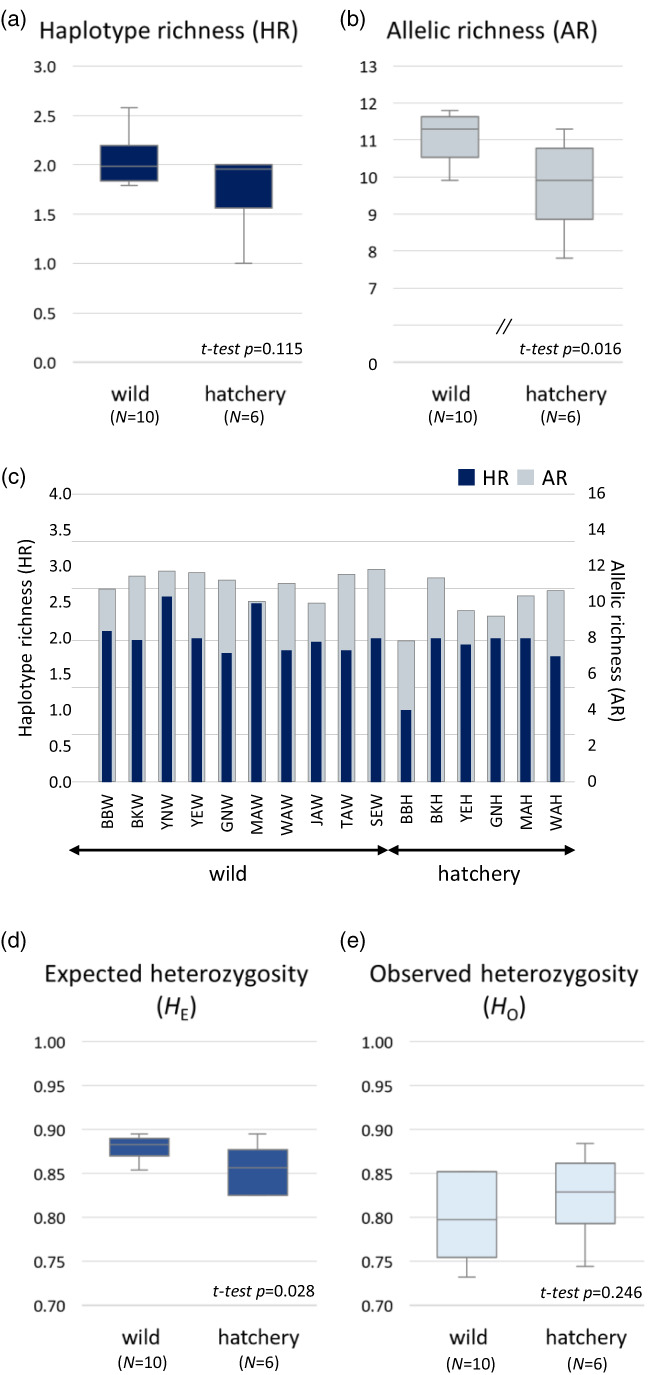
Comparisons of the levels of genetic diversity indices, including haplotype richness (HR) at mtDNA CR (a), allelic richness (AR) at ten microsatellite loci (b), and population‐level analyses (c), expected heterozygosity (*H*_E_) (d), and observed heterozygosity (*H*_O_) (e) between wild and hatchery populations. Only AR (b) and *H*_E_ (d) were significantly higher for wild than hatchery samples (AR: *t*‐test, *p* = 0.016; *H*_E_: *t*‐test, *p* = 0.028).

The mean levels of *H* and *π* for wild and hatchery populations were 0.454 ± 0.028 and 0.002 ± 0.002, and 0.493 ± 0.038 and 0.002 ± 0.002, respectively (Table 2). The HR level was higher for wild populations (mean HR = 2.053) than for hatchery populations (1.777), albeit not significantly so (independent *t*‐test, *df* = 14, *t* = 1.68, *p* = 0.115; Figure 3a). Microsatellite diversity (AR) was significantly higher for wild populations (mean AR = 11.1) than for hatchery populations (9.8) (independent *t*‐test, *df* = 14, *t* = 2.74, *p* = 0.016; Figure 3b,c). Similarly, levels of *H*_E_ and *H*_O_ tended to be higher in wild populations compared to hatchery ones, although only *H*_E_ showed a statistical significance (independent *t*‐test, *df* = 14, *t* = 2.44, *p* = 0.028; Figure 3d,e). The LD method gave median estimates of *N*_e_ of only 16.8 (BBh) to infinity (∞) (MAw and TAw; Table 2). Consistent with the microsatellite diversity differences, *N*_e_ estimates were significantly higher in wild populations than in hatchery populations (Mann–Whitney *U* test, *p* = 0.013; Table 2).

### 3.2 Genetic differentiation and structure among Korean populations

**FIGURE 4:**
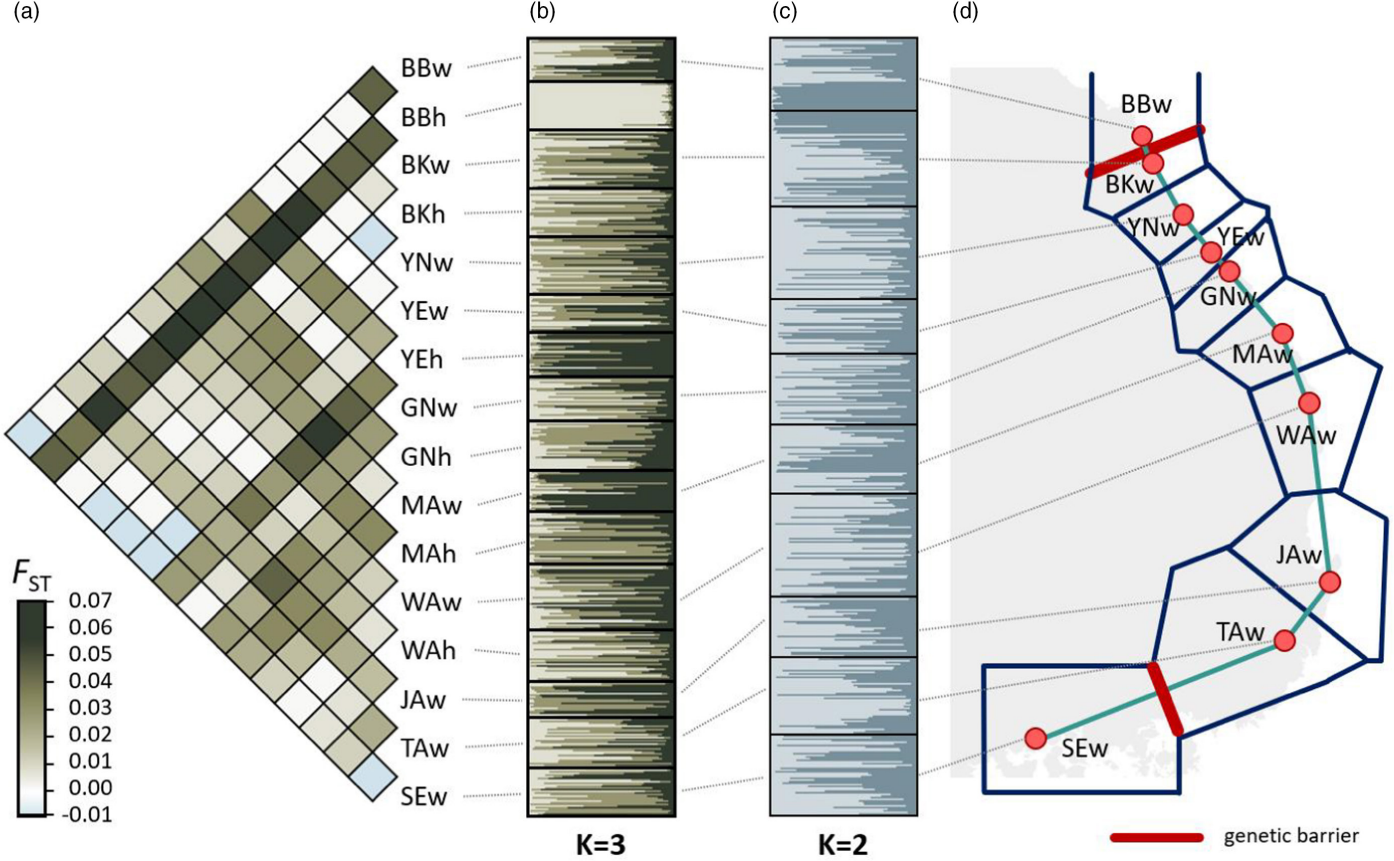
Population genetic structure of *Oncorhynchus keta* from South Korea. (a) Levels of genetic differentiation (*F*_ST_) among the 16 populations (including 10 wild and six hatchery samples). *F*_ST_ values are given in Table S2. Population abbreviations as in Table 1. Bayesian model‐based clustering test under an admixed ancestral population for the 16 populations (including 10 wild and six hatchery samples) responds to *K* = 3 (b) and only wild populations assignment test assuming *K* = 2 (c). Each color indicates different genetic clusters. (d) Genetic barrier inferred from gene flow patterns based on *F*_ST_ values of mtDNA and microsatellites. Thick red lines denote the first and second largest genetic barriers.

Temporal *F*_ST_ estimates for the three wild populations were not significant (mtDNA: *F*_ST_ for BKw = 0.059, YNw = 0.004, WAw = 0.070; microsatellites: *F*_ST_ for BKw = 0.029, YNw = 0.004, WAw = 0.012) except BKw at microsatellites. While only 19 of 120 pairwise population comparisons were significant for mtDNA CR (mean *F*_ST_ = 0.042), 79 pairwise *F*_ST_ estimates were significant among the 120 comparisons for microsatellites (mean *F*_ST_ = 0.020; Table S2; Figure 4a). These results suggest overall weak, but detectable local‐scale genetic differentiation among the Korean chum salmon populations at a 13~400 km geographic scale. The greatest levels of genetic differentiation were detected between BBh and SEw (*F*_ST_ = 0.261) and between BBh and YEh (*F*_ST_ = 0.070) for mtDNA and microsatellites, respectively. Based on mtDNA, all of the 19 significant pairwise *F*_ST_ statistics were involved with two particular hatchery samples (BBh and GNh), indicating that the remaining 14 populations are genetically homogeneous except for these two populations. By comparison, significant microsatellite *F*_ST_ values between wild populations always included MAw and JAw. No significant *F*_ST_ comparisons between populations included YNw, Yew, and SEw. Unexpectedly, two and six pairs of *F*_ST_ statistics between wild and hatchery populations within the same river basins revealed a significant divergence at mtDNA and microsatellites, respectively (mtDNA: BBw vs. BBh, GNw vs. GNh; microsatellites: BBw vs. BBh, BKw vs. BKh, YEw vs. YEh, GNw vs. GNh, MAw vs. MAh, WAw vs. WAh; Table S2). These results indicate strong genetic drift effects on hatchery samples over generation. Alternatively, hatchery populations might have been homogenized for the stocking practices, which could result in them being genetically distinct from local wild populations.

A first STRUCTURE analysis including entire samples calculated the highest Ln (*K*) and Δ*K* value that was *K* = 3. A ratio of three genotypes across all the populations was nearly equally distributed among clusters (30.3%, 35.9%, 33.8%; Figures 2b and 4b). In some populations, however, one of the three genotypes was predominant, for example, BBh (91.8%, 4.7%, 3.5%), YEh (10.3%, 17.9%, 71.8%), and MAw (12.5%, 16.3%, 71.1%). The second STRUCTURE analysis including only wild populations indicated that the most likely number of clusters was two (*K* = 2; Figure 4c). When hatchery samples were excluded, 10 wild populations showed relatively homogenous distributions of individual genotypes, in which all salmon individuals were assigned to approximately equal proportions of the inferred two genetic clusters, indicating their microsatellite gene pool is fairly admixed. Approximately, similar proportions of the inferred two genetic clusters suggest that all Korean wild salmon populations are genetically undifferentiated and so could not form genetic clusters, most likely due to the low level of *F*_ST_ values (overall mean *F*_ST_ = 0.020; Latch et al., 2006). Results of the population‐level PCA revealed that the first two principle component factors of PC1 and PC2 explain 27.7% and 20.9% of the total variation, respectively (Figure S1). The 13 populations were clustered together except for BBh (with reference to PC1) and YEh and GNh (PC2) in PCA.

Based on *F*_ST_ estimates of mtDNA and microsatellites with information on latitudes and longitudes for each locality, BBw and SEw were identified as a top of the two genetic barriers under the *K* = 2 scenario (Figure 4c,d). The findings suggest that the northernmost‐ and the southernmost populations may encounter very limited gene flow from more geographically central salmon populations (Eckert et al., 2008). It would thus be conceivable that edge effects could account for why the BBw and SEw populations are more genetically divergent, but larger *N*_e_ values for these populations (Table 2) are contrary to this explanation. Additional spatial genetic analysis, IBD detected a positive relationship between genetic (*F*_ST_) and geographical distances based on mtDNA, albeit not statistically significant (*r* ^2^ = 0.065, *p* = 0.15; Figure S2). The Mantel test for microsatellites also showed no significant association (*r* ^2^ = 0.043, *p* = 0.17; Figure S2).

The hierarchical AMOVA for both mtDNA and microsatellites showed a lack of genetic structure between wild and hatchery groups (mtDNA: *F*_CT_ = −0.006, *p* = 0.835; microsatellites: *F*_CT_ = 0.001, *p* = 0.059), but rather highly significant genetic structure among populations within groups (mtDNA: *F*_SC_ = 0.035, *p* < 0.001; microsatellites: *F*_SC_ = 0.021, *p* < 0.001; Table S3). Additionally, temporal AMOVA analyses at both mtDNA and microsatellites showed a lack of genetic structure among temporal samples (mtDNA: *F*_CT_ = −0.013, *p* = 0.425; microsatellites: *F*_CT_ = −0.003, *p* = 0.619; Table S3).

### 3.3 Genetic diversity and differentiation among North Pacific populations

**FIGURE 5:**
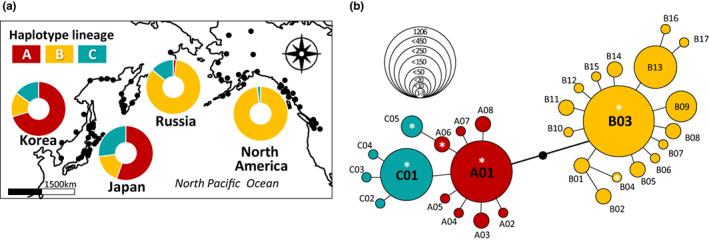
Global‐scale distribution of haplotype lineages (A, B, C) and their proportions in the four regional populations (Korea, Japan, Russia, and North America) of chum salmon, *Oncorhynchus keta*. Haplotype distribution (a) and haplotype network (b) of three matrilineal lineages in the North Pacific Ocean, based on 30 haplotypes of mtDNA control region (481 bp). Six haplotypes (A01, A06, B03, B04, C01, and C05) represented by asterisks (*) indicate the haplotypes detected in this study.

The observed 30 CR haplotypes (481 bp) were grouped into three main matrilineal lineages (A: 27.8%, B: 59.8%, C: 12.4%; Figure 5a) in 2511 individuals including 2162 from the previous study (Sato et al., 2004) and 349 individuals in this study. Compared with Russian (A: 1.7%, B: 84.2%, C: 14.0%) and North American lineages (A: 0.0%, B: 99.5%, C: 0.5%), the Korean lineage (A: 72.4%, B: 14.7%, C: 12.9%) consisted of similar frequencies as the Japanese one (A: 55.5%, B: 17.4%, C: 27.1%), although the proportion of lineage A was 16.9% higher in the Korean population. These results suggest that while lineage B has evolved predominantly in regions of Russia and North America, lineage A has diversified in Korea and Japan. The three major lineages in the North Pacific Rim included only 30 haplotypes, which were linked by only one to six mutational steps, indicative of shallow evolutionary history (Figure 5b).

**FIGURE 6:**
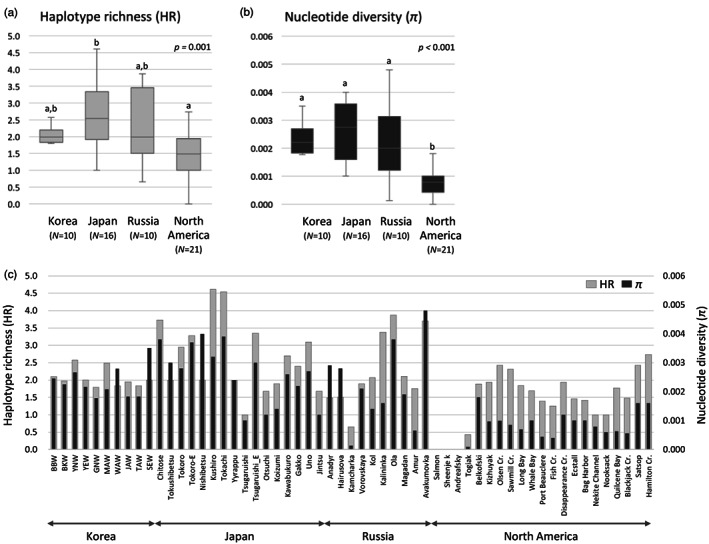
Comparisons of the level of genetic diversity, including haplotype richness (HR) (a), nucleotide diversity (π) (b), and population‐level analyses (57 populations) (c) of mtDNA control region among the four regional populations (Korea, Japan, Russia, North America) in the North Pacific Ocean. Statistical significance at *p* < 0.05 is indicated with upper case letters for the differences of HR (a) and π (b).

With respect to the level of genetic diversity, while the entire Korean population showed the lowest HR value, Japanese and Russian populations had relatively higher levels of mtDNA diversities (Table 3). HR values for entire pooled Korean, Japanese, Russian, and North American populations were 5.00, 11.05, 11.42, and 8.20, respectively (Table 3). At the population level, however, mean HR and *π* values were the second highest for the Korean salmon (mean HR = 2.05, mean *π* = 0.002; Figure 6). Mean values of HR, H, and *π* in Japanese populations were 2.68, 0.607, and 0.003, respectively, which were the highest levels among regional populations (Figure 6). Russian populations showed the highest value of mean HR per population (2.24). At a regional scale, Korean populations had the lowest diversity in terms of HR (5.00), which showed more than two‐fold lower than Japanese populations.

**TABLE 3:** Measures of genetic diversity and divergence for each of the North Pacific regional population of *Oncorhynchus keta* based on mtDNA control region (CR) sequences after rarefaction to a common sample size of Korean population (*N* = 395 [*N* = 349 in this study and *N* = 46 from Sato et al., 2004]).

Region	*N*	*N*_H_	*H* (SE)	HR	*H*_S_	*H*_T_	*G*_ST_	*C*_T_	*C*_S_	*C*_D_	$C_{T}^{r}$	$C_{S}^{r}$	$C_{D}^{r}$
Korea	395	6	0.454 (0.028)	5.00	0.464	0.772	0.399	0.045	−0.007	0.052	−0.091	−0.094	0.003
Japan	731	16	0.633 (0.013)	11.05	0.524	0.758	0.309	0.025	0.074	−0.049	0.123	0.051	0.072
Russia	406	13	0.447 (0.029)	11.42	0.462	0.706	0.346	−0.045	−0.01	−0.035	0.153	0.06	0.093
North America	979	11	0.342 (0.018)	8.20	0.427	0.721	0.408	−0.025	−0.057	0.032	0.149	−0.017	0.167

Abbreviations: *C*_T_, *C*_S_, *C*_D_, contribution indices to total species‐level diversity; $C_{T}^{r}$, $C_{S}^{r}$, $C_{D}^{r}$, contribution indices to total haplotype richness; HR, haplotype richness; *H*_S_, *H*_T_, *G*_ST_, divergence indices from the other populations; *N*, number of specimens; *N*_H_, number of haplotypes; *H* (SE), haplotype diversity with standard error in brackets.

The divergence of the populations showed that genetic (haplotype) differentiation was the largest for North America (*G*_ST_ = 0.408) followed by Korea (0.399) located at the south‐western geographical limits (Table 3). The Korean population contributed more significantly to the total species‐level genetic diversity, as indicated by the highest positive *C*_T_ (0.045), which was mostly due to the strong divergence (the distinctiveness contribution; *C*_D_ = 0.052) of the Korea population from the other regional ones (Table 3). Negative *C*_D_ values for Japanese and Russian populations represented a lack of significant differentiation among regional populations. However, Korean population genetically differed from the three regional populations, as shown in significant values of pairwise *F*_ST_ (Korea vs. Japan: *F*_ST_ = 0.026; Korea vs. Russia: 0.533; Korea vs. North America: 0.762; Table S4), indicating that the East Sea population in Korea has diversified and evolved a unique genetic identity, which highlights the fact that conservation of this unique genetic variation should be a priority. AMOVA further supported the findings of regional‐scale genetic divergence among the four geographic populations (*F*_CT_ = 0.588, *p* < 0.001; Table S5).

Eight microsatellite markers also showed the similar patterns of the lowest level of diversity in Korean populations (Kruskal–Wallis test, *p* < 0.001), although unequal sample sizes could not be corrected among the four regional populations (number of individuals; Korea: *N* = 272; Japan: *N* = 2729; Russia; *N* = 2923; North America: *N* = 6482; Figure S3).

## 4 DISCUSSION

The chum salmon *O. keta* serves as an important fishery resource around the North Pacific Ocean and also plays a pivotal role in sustaining biodiversity and ecosystem health in freshwater habitats as a keystone species (Willson & Halupka, 1995). Although a handful of studies have assessed genetically the North Pacific populations of *O. keta*, the southwestern marginal populations from the Korean Peninsula in the species' distributional range were largely overlooked except for one, the largest Korean population (YNw; the Yangyang Namdae Stream). To our best knowledge, this study is the first to explore the “local‐scale” genetic structure of the salmon populations spanning almost its entire geographical range in South Korea from the Seomjin River (SEw; the southernmost limit; Figure 2) on the South Sea to the Baebong Stream (BBw; the northern limit) on the East Sea in the Korean Peninsula. By analyzing mtDNA and microsatellites together, we further evaluate the genetic variation within/among wild (*N* = 10) and hatchery (*N* = 6) populations of the Korean chum salmon, comprising its geographically peripheral populations at the southwestern distributional edges. Based on the results of the comparative analysis of levels of genetic diversity and divergence with other regional populations distributed across its entire geographical range, we show that Korean *O. keta* populations house the unique genetic integrity representing their own evolutionary history or trajectory, with a relatively lower genetic diversity but greater contribution to overall species‐level diversity (Table 3). In general, the range‐edge population is predicted to show reduced genetic diversity as well as elevated population differentiation with a lower *N*_e_ and restricted gene flow than the center of species distribution (Eckert et al., 2008; Sagarin & Gaines, 2002). Knowledge of how genetic diversity is distributed across species' ranges now becomes recognized as an important issue particularly considering climate change from a conservation perspective (Gibson et al., 2009). We here thus highlight and advocate for the ecological or evolutionary significance of the Korean populations of chum salmon from the species‐level conservation and management perspectives.

The lowest mtDNA diversity (HR = 5.00) with a relatively larger contribution of Korean populations to the species‐level genetic diversity advocates safeguarding their diversity and gene pool with a high priority (Consuegra et al., 2005). The Korean population's larger role in shaping the overall genetic diversity in chum salmon highlights that we should pay more attention on how the “local‐scale” genetic diversity sometimes disproportionally represents the global diversity particularly in highly dispersing species such as *O. keta* (Lee & Boulding, 2009). A previous study of Atlantic salmon *Salmo salar* showed that high levels of asymmetric gene flow from geographically central populations may help to prevent from reduction in genetic diversity in the peripheral populations in the Iberian Peninsula (Consuegra et al., 2005). Microsatellite markers showed the similar patterns of the lowest level of diversity in Korean populations, albeit unequal sample sizes among the regional populations, when analyzing the same maker‐set of eight loci analyzed (Figure S3). Note that microsatellite scoring may not be consistent across different laboratories. In a previous study of Sato et al. (2004), the Korean population (YNw) showed considerably lower genetic diversity (*H* = 0.37) in mtDNA CR relative to entire North Pacific populations (*H* = 0.63). We now find that the level of HR in Korean populations was 5.00, which is the lowest mtDNA diversity among the regions (Japan = 11.05, Russia = 11.42, North America = 8.20; Table 3), although there is a difference in sampling periods, particularly for the Korean populations sampled 20~30 years more recent in this study. Our study further shows that the Korean salmon population is genetically divergent from other regional populations and even with Japanese one (*F*_ST_ = 0.026, *p* < 0.01) based on mtDNA CR. These findings highlight its own “genetic integrity” of the Korean population with reference to other regional ones. In the case of long‐range migrating organisms like chum salmon, local‐scale genetic diversity and population structure are often neglected for understanding their genetic status. However, our findings underline there is a need to pay more attention to the Korean salmon population, which has continually decreased in the population size and returning adults (NPAFC, 2018, 2019). Given intraspecific genetic diversity is well documented to be associated with *N*_e_ and adaptive potential (Frankham, 1996), genetically isolated populations of Korean salmon with a relatively lower diversity (a smaller *N*_e_) are expected to be more susceptible to genetic drift effects causing the loss of unique and rare haplotypes/alleles unless gene flow is overwhelming. Our estimated mean value of median *N*_e_ for eight wild populations (without considering two populations from MAw and TAw having infinity [∞]) is approximately 373, which may provide evidence supporting the hypothesis of low genetic diversity with a small *N*_e_ for the Korean salmon population (Table 2). The reduced genetic diversity eventually diminishes the evolutionary potential of the Korean salmon population to adapt to changes in environmental conditions, such as ongoing climate changes with rising sea surface temperature (Franks & Hoffmann, 2012).

Expectedly, the levels of mtDNA and microsatellite diversities (HR, AR) tend to be greater for wild than for hatchery samples, although only AR was statistically significant. Several countries including South Korea exploited the artificial propagation of wild salmonid adults by captive breeding and rearing juveniles in hatchery environments, and then releasing back to their natal rivers, for the purpose of the conservation and management programs (Seong, 1998). Stocking management practice of Korean chum salmon started in the 1970s, when the eggs were introduced from United States of America (USA) and Japan (Myoung et al., 1993). The B clade (Figure 5) might have been introduced and established in the Korean population through this stocking activity. Despite the enormous efforts, many hatchery populations are challenged by the loss of within‐population genetic diversity due to genetic drift and founder effects, which are the direct outcomes of these stocking activities (Aho et al., 2006; Setzke et al., 2022), despite some exceptions (e.g., Eldridge & Killebrew, 2008). The low diversity in hatchery fish may in turn lead to fitness declines in the wild over a very short period of captive rearing even in only one or two generations (Araki et al., 2008; Utter, 1998). Hatchery‐reared fish generally show a poorer ability in predator avoidance and has a lower breeding success in the wild (Araki et al., 2007). Therefore, the use of hatchery fish as an effective means of stock enhancement for salmonids has been hotly debated (Araki & Schmid, 2010). It is perhaps necessary to improve current hatchery stocking programs by monitoring genetic diversity and structure in both the hatchery stocks and progeny, and controlling inbreeding within the stocks to maintain considerable levels of genetic diversity. Microsatellite‐based parentage analysis will also be useful for the evaluation of reproductive fitness in the wild (Araki & Schmid, 2010).

We find weak, but detectable local‐scale genetic differentiation among the Korean chum salmon wild populations at a 13 to 400‐km geographic scale. Cautiously, there is detectable genetic divergence between some wild and the respective hatchery populations. The observed significant differentiation between the wild and hatchery stocks might result from the reduced genetic diversity in hatchery samples due to a founder effect (Yu et al., 2012) or from the use of nonlocal origin for the broodstock, meaning that geographically or genetically divergent populations were historically introduced into hatchery brood stocks (Nielsen et al., 1994). The use of admixed hatchery juvenile salmons originated from different natal streams for releasing practice can also contribute to the observed genetic divergence between wild and hatchery populations. Therefore, we should be cautious to only stock hatchery individuals from the same rivers for stocking on wild populations. Genetic assessments should be performed before releasing hatchery‐reared juveniles back to the wild.

However, overall genetic composition of the Korean chum salmon population can be regarded as well‐admixed population structure. Weak population structure or genetic homogeneity among 16 populations could be explained by several plausible hypotheses: (1) introgressive hybridization and/or bottleneck with hatchery‐reared conspecifics (Östergren et al., 2021; Ozerov et al., 2016); (2) local populations at small geographic scales over a very large distributional range, which might have originated from a shared recent evolutionary ancestry; and (3) the limited number of microsatellite markers used, although they have high number of alleles. The first hypothesis is that inadvertent gene flow via releasing captive‐bred fish from different rivers into natural environments causes homogenizing genotypic compositions across populations (Chapman et al., 2002; Marie et al., 2010; Ozerov et al., 2016). Many salmon hatcheries typically use returning adults for spawning by combining eggs from several females and milt from several males in a single container. In Korea, many juveniles hatched from their parents of each stream, are mix‐reared in the FIRA (BBh, BKh, YEh, and GNh), GFFRC (WAh), and SAFRC (MAh), and then released into several streams during the next spring. Especially, hatchery‐raised juvenile salmons from the Yangyang Namdae Stream (YNw) were predominantly (approximately 70% of entire juveniles) used for the release in a majority of rivers, particularly for the populations of BBw, BKw, YNw, Yew, and GNw, which is highly likely to homogenize the locally distinguishable genetic structure if exists. Through these stocking practices, inadvertent introgressive hybridization across the populations would be unavoidable. As a consequence, they are more threatened by genetic homogenization such as loss of local adaptation from indigenous gene pools according to these anthropogenic interferences (Ozerov et al., 2016). In the case of the Red drum (*Sciaenops ocellatus*) from South Carolina along the Atlantic Coast of the United States, the level of genetic differentiation among populations has gradually decreased from *F*_ST_ = 0.319 to 0.028 with increasing time intervals over 5 years by hatchery releasing program (Chapman et al., 2002). Now, after more than 40 years from stock activities started in the Yangyang Inland Fisheries Research Institute, one of the factors erasing genetic differences between wild and hatchery salmons and also between wild salmons may be linked to the genetic admixture by extensive stocking (Seong, 1998).

A second hypothesis is that a lack of genetic structure among 10 wild Korean populations is attributed to the fact that spatial scales analyzed across the eastern coast of Korea is particularly small, considering chum salmon's entire geographic range. *Oncorhynchus keta* is known to have originated from two evolutionary lineages, Western (East Asia) and Eastern (North America) of the North Pacific (Beacham et al., 2009; Sato et al., 2004; Seeb et al., 2011). Three kinds of genetic markers (e.g., mtDNA CR, microsatellites and SNPs) previously analyzed indicate four different genetic clusters, such as Korea/Japan, Russia, Alaska, and North America. According to Beacham et al. (2009), a Korean population could be identified as a single unique lineage because it is evolutionarily distinct from Japanese populations in Hokkaido and Honshu. Furthermore, several studies of chum salmon revealed the population divergence detected only over a geographic distance (coastline distance) of at least 600 km among populations in Russia (Afanas'ev et al., 2011). Adjacent populations in Hokkaido and Honshu Island from Japan (within 50 km) showed no genetic differentiation (Sato et al., 2001). In the case of Alaska and Canada belonging to the Eastern North Pacific group, *F*_ST_ estimates between neighboring streams within Norton Sound Drainage (40–600 km) were not significant except for a few stream pairs (mean *F*_ST_ = 0.003, mean *p* = 0.106; Olsen et al., 2008). In our study, the coastline distance among populations ranges from 18 km (BBw and BKw) to 400 km (BBw and SEw). At an even much smaller geographic scale, our study finds weak but some cases of detectable genetic differentiation across local populations of *O. keta* from South Korea.

Lastly, microsatellite loci used in this study may not have sufficient resolution for uncovering genetic differentiation among the Korean populations. However, 53 SNPs developed for chum salmon have also shown a single genetic cluster from Korean and Japanese populations, although only the Yangyang Namdae Stream (YNw) was considered for the Korean population (Seeb et al., 2011). Nevertheless, further analysis at the whole genome level or a much a higher number of molecular markers along the genome (e.g., microsatellites) might be necessary to reach a firm conclusion on whether Korean population is comprised of genetically separate populations.

In conclusion, the observed lower genetic diversity and unique genetic integrity of the Korean chum salmon population at the species range margin highlight an urgent need for conservation efforts with a high priority. Although abundances of commercial catch and the level of genetic diversity of *O. keta* are still moderately high across a whole species range of chum salmon at a global scale, some local populations such as Korea, Japan, Amur River, western Alaska, and Columbia River are now under severe threat of local extinction (Ruggerone et al., 2010). Thus, we here argue that for the purpose of the chum salmon conservation and management, the Korean population should be paid more attention to.

At a local scale, we should be cautious about the single gene pool hypothesis of the Korean populations, given some detectable local differentiation also present, although overall well‐admixed gene pool is likely. We also argue that sustaining the unique haplotypes observed in some local populations (e.g., BBw at the northern limit) will help to preserve locally adapted gene pool. For this, it would be more appropriate to use juvenile individuals born from their home streams for releasing practice on the respective wild populations. To increase or restore the level of genetic diversity for some Korean populations, breeding individuals from particular streams with high (e.g., YNw, SEw) and low (BBh) genetic diversity would be plausible. Most importantly, a continuous genetic monitoring is crucial for understanding how well these valuable populations will maintain under ongoing climate changes.

## CONFLICT OF INTEREST

The authors declare that they have no known competing financial interests or personal relationships that could have appeared to influence the work reported in this paper.

## Supporting information


Figure S1

Figure S2

Figure S3

Table S1

Table S2

Table S3

Table S4

Table S5

## Data Availability

The mtDNA control region sequences of six haplotypes obtained for this study have been deposited in GenBank under the accession numbers ON989345–ON989350. The datasets of microsatellite genotypes are available on Dryad digital repository (https://doi.org/10.5061/dryad.w0vt4b8vc).
