# Supplementary figures and images for: Low genetic diversity, local‐scale structure, and distinct genetic integrity of Korean chum salmon (Oncorhynchus keta) at the species range margin suggest a priority for conservation efforts
Source: Evol Appl. 2022 Nov 10;15(12):2142–57. doi: 10.1111/eva.13506 (PMC9753833; doi:10.1111/eva.13506)

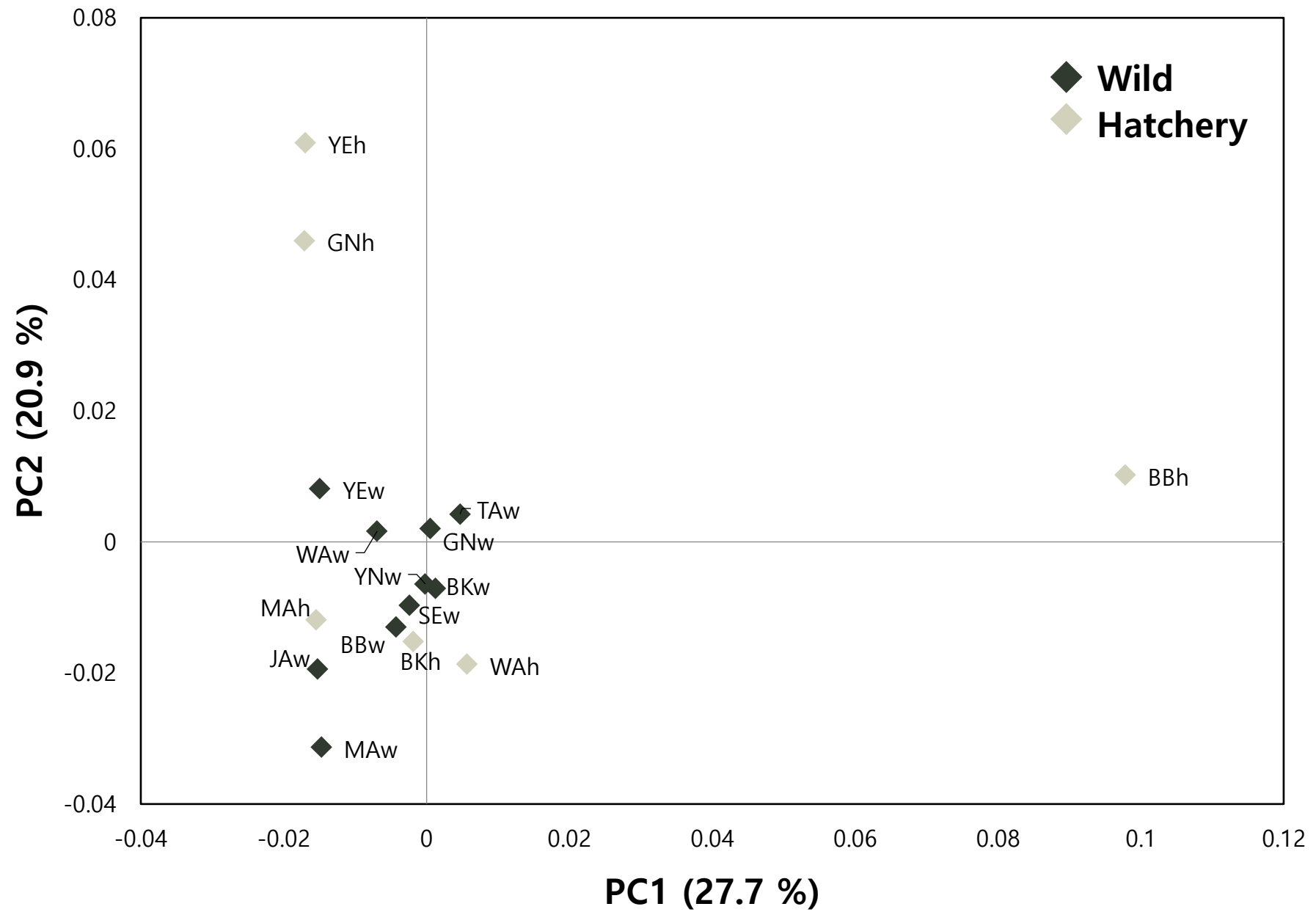

Supplement: Supplementary file 1 — Figure S1 [file EVA-15-2142-s002.pdf]

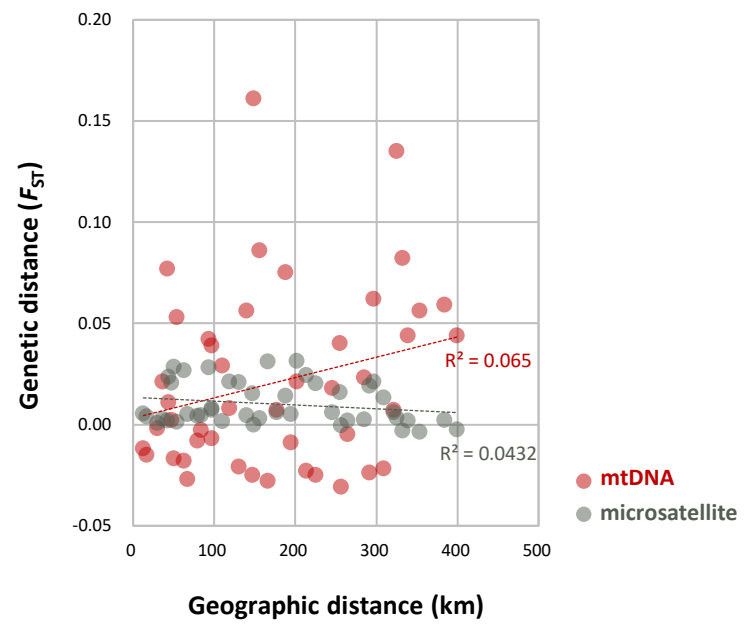

Supplement: Supplementary file 2 — Figure S2 [file EVA-15-2142-s005.pdf]

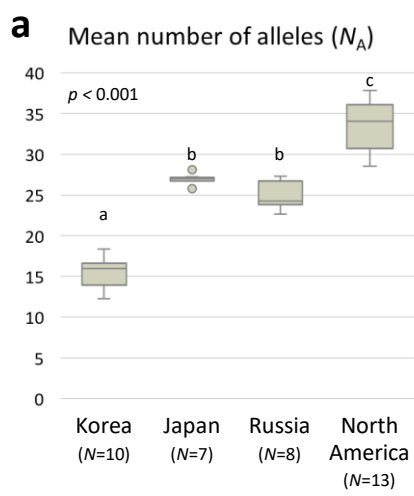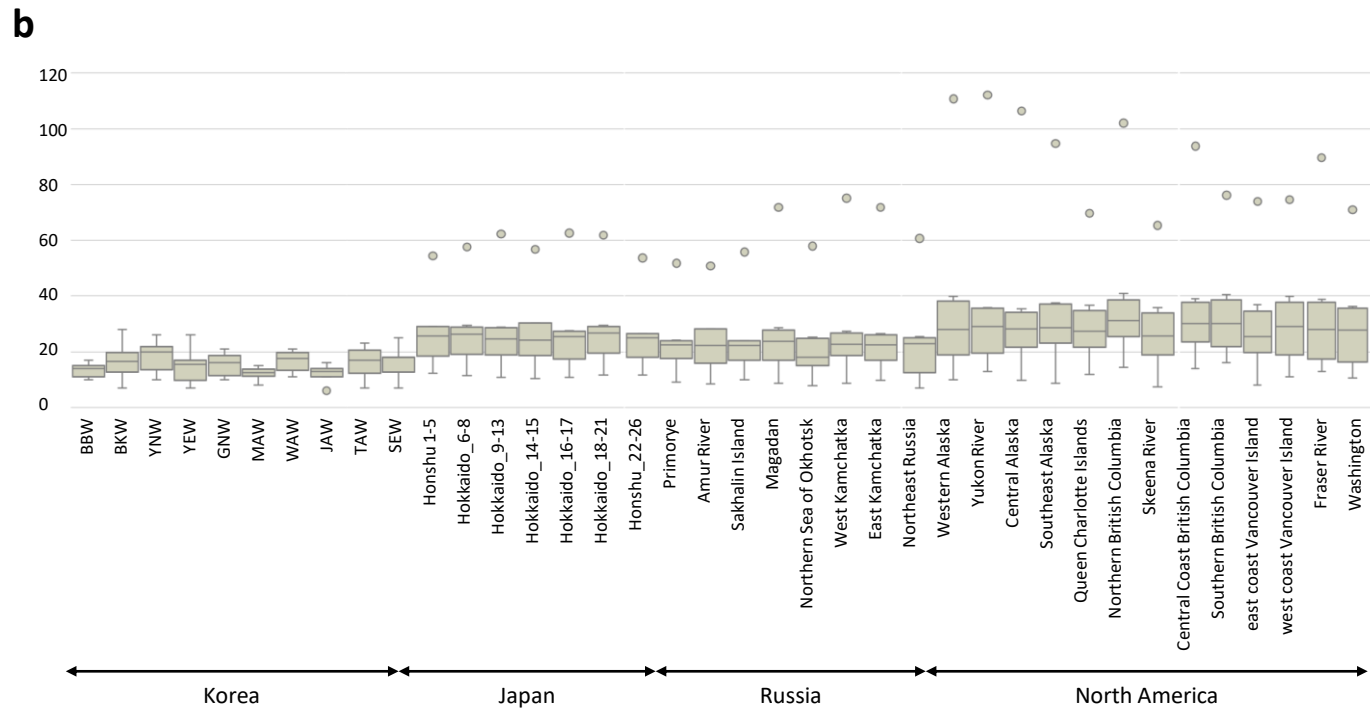

Supplement: Supplementary file 3 — Figure S3 [file EVA-15-2142-s008.pdf]
