# Supplementary material for: Low genetic diversity, local‐scale structure, and distinct genetic integrity of Korean chum salmon (Oncorhynchus keta) at the species range margin suggest a priority for conservation efforts
Source: Evol Appl. 2022 Nov 10;15(12):2142–57. doi: 10.1111/eva.13506 (PMC9753833; doi:10.1111/eva.13506)
Supplement: Supplementary file 4 — Table S1 [file EVA-15-2142-s004.docx]

**Table S1** Information of mtDNA control region (CR) sequences used for constructing haplotype network based on previous studies. Haplotype IDs, A01 to A08, B01 to B17, and C01 to C05, which were found in previous studies (Sato et al., 2001, 2004) were renamed in this study. The haplotype IDs are the same as in Figure 5. Six haplotypes (A01, A06, B03, B04, C01, C05) detected in this study are highlighted in grey.

| **Renamed haplotype IDs**  **(in this study)** | **Lineages** | **Original haplotype name** | **GenBank nos.** | **References** |
| --- | --- | --- | --- | --- |
| A01 | A-1 | OKDL-1 | AB039890 | Sato et al. 2001 |
| A02 | A-2 | A-2 | AB091514 | Sato et al. 2004 |
| A03 | A-3 | A-3 | AB091515 | Sato et al. 2004 |
| A04 | A-4 | A-4 | AB091516 | Sato et al. 2004 |
| A05 | A-5 | OKDL-2 | AB039891 | Sato et al. 2001 |
| A06 | A-6 | OKDL-3 | AB039892 | Sato et al. 2001 |
| A07 | A-7 | OKDL-4 | AB039893 | Sato et al. 2001 |
| A08 | A-8 | OKDL-5 | AB039894 | Sato et al. 2001 |
| B01 | B-1 | OKDL-6 | AB039895 | Sato et al. 2001 |
| B02 | B-2 | B-2 | AB091517 | Sato et al. 2004 |
| B03 | - | OKD4 | AB524890 | Unpublished |
| B04 | B-4 | OKDL-8 | AB039897 | Sato et al. 2001 |
| B05 | B-5 | B-5 | AB091518 | Sato et al. 2004 |
| B06 | B-6 | B-6 | AB091519 | Sato et al. 2004 |
| B07 | B-7 | B-7 | AB091520 | Sato et al. 2004 |
| B08 | B-8 | B-8 | AB091521 | Sato et al. 2004 |
| B09 | B-9 | B-9 | AB091522 | Sato et al. 2004 |
| B10 | B-10 | B-10 | AB091523 | Sato et al. 2004 |
| B11 | B-11 | B-11 | AB091524 | Sato et al. 2004 |
| B12 | B-12 | B-12 | AB091525 | Sato et al. 2004 |
| B13 | B-13 | B-13 | AB091526 | Sato et al. 2004 |
| B14 | B-14 | B-14 | AB091527 | Sato et al. 2004 |
| B15 | B-15 | B-15 | AB091528 | Sato et al. 2004 |
| B16 | B-16 | B-16 | AB091529 | Sato et al. 2004 |
| B17 | B-17 | B-17 | AB091530 | Sato et al. 2004 |
| C01 | C-1 | OKDL-9 | AB039898 | Sato et al. 2001 |
| C02 | C-2 | OKDL-10 | AB039899 | Sato et al. 2001 |
| C03 | C-3 | C-3 | AB091531 | Sato et al. 2004 |
| C04 | C-4 | OKDL-11 | AB039900 | Sato et al. 2001 |
| C05 | C-5 | OKDL-12 | AB039901 | Sato et al. 2001 |
