# Supplementary material for: Low genetic diversity, local‐scale structure, and distinct genetic integrity of Korean chum salmon (Oncorhynchus keta) at the species range margin suggest a priority for conservation efforts
Source: Evol Appl. 2022 Nov 10;15(12):2142–57. doi: 10.1111/eva.13506 (PMC9753833; doi:10.1111/eva.13506)
Supplement: Supplementary file 5 — Table S2 [file EVA-15-2142-s001.docx]

**Table S2** Pairwise genetic differentiation (*F*_ST_) based on mtDNA control region sequences (above diagonal) and ten microsatellites loci genotypes (below diagonal) for 16 populations of *Oncorhynchus keta* from South Korea. Significant pairwise *F*_ST_ and *P* values are bolded (*P* < 0.05) after the Bonferroni correction. The *F*_ST_ values highlighted in grey represent significant differentiation between wild and hatchery populations within the same river basins.

|  | BBw | BBh | BKw | BKh | YNw | YEw | YEh | GNw | GNh | MAw | MAh | WAw | WAh | JAw | TAw | SEw |
| --- | --- | --- | --- | --- | --- | --- | --- | --- | --- | --- | --- | --- | --- | --- | --- | --- |
| BBw |  | **0.139** | -0.003 | 0.033 | 0.065 | -0.006 | -0.029 | -0.013 | **0.018** | 0.006 | -0.026 | -0.013 | 0.013 | 0.022 | 0.027 | 0.087 |
| BBh | **0.043** |  | **0.093** | 0.134 | 0.040 | 0.043 | 0.127 | **0.125** | **0.129** | **0.132** | 0.118 | **0.161** | **0.171** | **0.137** | 0.029 | 0.261 |
| BKw | 0.002 | **0.041** |  | 0.012 | 0.015 | -0.029 | -0.008 | -0.007 | **0.065** | -0.019 | 0.007 | 0.006 | 0.003 | -0.017 | -0.001 | 0.081 |
| BKh | 0.002 | **0.044** | 0.003 |  | 0.004 | 0.018 | 0.039 | 0.067 | **0.073** | 0.019 | 0.033 | 0.005 | 0.090 | 0.026 | 0.065 | -0.019 |
| YNw | 0.002 | **0.041** | 0.002 | -0.001 |  | -0.002 | 0.062 | 0.069 | **0.099** | 0.028 | 0.062 | 0.061 | 0.085 | 0.027 | 0.021 | 0.074 |
| YEw | 0.004 | **0.051** | 0.004 | -0.001 | 0.001 |  | -0.016 | -0.020 | 0.042 | -0.016 | -0.006 | 0.014 | -0.001 | -0.014 | -0.033 | 0.101 |
| YEh | **0.031** | **0.070** | **0.028** | **0.032** | **0.026** | **0.020** |  | -0.023 | **0.014** | 0.005 | -0.030 | -0.008 | 0.006 | 0.020 | 0.013 | 0.104 |
| GNw | 0.009 | **0.046** | 0.004 | 0.010 | 0.002 | 0.005 | **0.029** |  | **0.055** | 0.000 | -0.003 | 0.019 | -0.017 | 0.009 | 0.000 | 0.156 |
| GNh | **0.027** | **0.067** | **0.028** | **0.031** | **0.030** | **0.018** | **0.039** | **0.029** |  | **0.101** | -0.021 | **0.032** | **0.118** | **0.124** | **0.071** | 0.125 |
| MAw | **0.013** | **0.065** | **0.016** | **0.018** | **0.022** | 0.020 | **0.049** | **0.023** | **0.045** |  | 0.030 | 0.013 | -0.006 | -0.027 | 0.016 | 0.084 |
| MAh | **0.013** | **0.062** | **0.018** | **0.012** | **0.013** | **0.014** | **0.043** | **0.027** | **0.033** | **0.029** |  | -0.010 | 0.043 | 0.049 | 0.025 | 0.087 |
| WAw | 0.002 | **0.044** | 0.005 | 0.002 | 0.002 | 0.001 | **0.024** | 0.007 | **0.019** | **0.015** | **0.013** |  | 0.045 | 0.027 | 0.056 | 0.030 |
| WAh | 0.011 | **0.042** | **0.006** | **0.003** | 0.007 | **0.010** | **0.039** | **0.011** | **0.035** | **0.023** | **0.015** | 0.006 |  | -0.001 | 0.018 | 0.184 |
| JAw | **0.012** | **0.064** | **0.018** | **0.018** | **0.015** | **0.018** | **0.044** | **0.025** | **0.039** | **0.030** | **0.031** | **0.017** | **0.017** |  | 0.017 | 0.099 |
| TAw | 0.002 | **0.038** | 0.005 | 0.001 | 0.002 | -0.001 | **0.028** | 0.006 | **0.023** | **0.024** | **0.018** | 0.002 | **0.009** | **0.023** |  | 0.174 |
| SEw | -0.002 | **0.041** | 0.001 | -0.003 | -0.003 | -0.004 | **0.027** | 0.002 | **0.027** | **0.015** | **0.013** | 0.000 | 0.006 | 0.014 | 0.000 |  |
