# Supplementary material for: Low genetic diversity, local‐scale structure, and distinct genetic integrity of Korean chum salmon (Oncorhynchus keta) at the species range margin suggest a priority for conservation efforts
Source: Evol Appl. 2022 Nov 10;15(12):2142–57. doi: 10.1111/eva.13506 (PMC9753833; doi:10.1111/eva.13506)
Supplement: Supplementary file 6 — Table S3 [file EVA-15-2142-s003.docx]

**Table S3** Hierarchical analysis of molecular variance (AMOVA) for the 16 populations including ten wild and six hatcheries (above) and also for temporal samples between 2017 and 2019 years of *Oncorhynchus keta* based on mtDNA control region (CR) and ten microsatellite loci. The temporal AVOVAs were analyzed by grouping all wild populations according to the sampling years (e.g. 2017FJ vs. 2017ADT & 2018FJ vs. 2018ADT & 2019 FJ; see Table 1).

| **Locus** | **Source of variation** | **df** | **Sum of**  **squares** | **Variance**  **components** | **% Variation** | **Fixation**  **indices** | **P** |
| --- | --- | --- | --- | --- | --- | --- | --- |
| **mtDNA** | Between groups (wild vs. hatchery) | 1 | 0.346 | ‒0.003 | ‒0.61 | *F*_CT_ = ‒0.006 | 0.835 |
|  | Among populations within groups | 14 | 15.017 | 0.018 | 3.56 | *F*_SC_ = 0.035 | < 0.001 |
|  | Within populations | 498 | 245.685 | 0.491 | 97.05 | *F*_ST_ = 0.029 | < 0.001 |
| **microsatellites** | Between groups (wild vs. hatchery) | 1 | 11.974 | 0.005 | 0.10 | *F*_CT_ = 0.001 | 0.059 |
|  | Among populations within groups | 14 | 136.273 | 0.093 | 2.09 | *F*_SC_ = 0.021 | < 0.001 |
|  | Within populations | 467 | 3998.596 | 4.354 | 97.80 | *F*_ST_= 0.022 | < 0.001 |
| **mtDNA** | Between temporal groups | 2 | 2.193 | ‒0.007 | ‒1.25 | *F*_CT_ = ‒0.013 | 0.425 |
|  | Among populations within groups | 3 | 6.630 | 0.019 | 3.68 | *F*_SC_ = 0.036 | 0.003 |
|  | Within populations | 533 | 269.462 | 0.506 | 97.57 | *F*_ST_ = 0.024 | <0.001 |
| **microsatellites** | Between temporal groups | 2 | 18.195 | ‒0.014 | ‒0.32 | *F*_CT_ = ‒0.003 | 0.619 |
|  | Among populations within groups | 3 | 28.006 | 0.046 | 1.04 | *F*_SC_ = 0.010 | 0.021 |
|  | Within populations | 956 | 4100.642 | 4.417 | 99.29 | *F*_ST_= 0.007 | 0.009 |
