# Supplementary material for: Low genetic diversity, local‐scale structure, and distinct genetic integrity of Korean chum salmon (Oncorhynchus keta) at the species range margin suggest a priority for conservation efforts
Source: Evol Appl. 2022 Nov 10;15(12):2142–57. doi: 10.1111/eva.13506 (PMC9753833; doi:10.1111/eva.13506)
Supplement: Supplementary file 7 — Table S4 [file EVA-15-2142-s006.docx]

**Table S4** Pairwise genetic differentiation (*F*_ST_) based on mtDNA control region sequences for four regional populations (Korea, Japan, Russia and North America) of chum salmon (*Oncorhynchus keta*) in the North Pacific Ocean. All pairwise *F*_ST_ and *P* values were significant (*P* < 0.01) after the Bonferroni correction.

|  | Korea | Japan | Russia |
| --- | --- | --- | --- |
| Japan | 0.026 |  |  |
| Russia | 0.533 | 0.502 |  |
| North America | 0.762 | 0.726 | 0.157 |
