# Supplementary material for: Low genetic diversity, local‐scale structure, and distinct genetic integrity of Korean chum salmon (Oncorhynchus keta) at the species range margin suggest a priority for conservation efforts
Source: Evol Appl. 2022 Nov 10;15(12):2142–57. doi: 10.1111/eva.13506 (PMC9753833; doi:10.1111/eva.13506)
Supplement: Supplementary file 8 — Table S5 [file EVA-15-2142-s007.docx]

**Table S5** Hierarchical analysis of molecular variance (AMOVA) for four regional groups (Korea, Japan, Russia and North America) including 57 populations of *Oncorhynchus keta* from the North Pacific based on mtDNA control region (CR).

| **Source of variation** | **df** | **Sum of**  **squares** | **Variance**  **components** | **% Variation** | **Fixation**  **indices** | **P** |
| --- | --- | --- | --- | --- | --- | --- |
| Among regional groups | 3 | 1181.707 | 0.674 | 58.84 | *F*_CT_ = 0.588 | < 0.001 |
| Among populations within regional groups | 53 | 138.098 | 0.051 | 4.41 | *F*_SC_ = 0.107 | < 0.001 |
| Within populations | 2408 | 1012.922 | 0.421 | 36.75 | *F*_ST_ = 0.633 | < 0.001 |
